# Homogeneous oxidation of SO_2_ in the tail gas incinerator of sulfur recovery unit

**DOI:** 10.1186/s13065-023-01096-w

**Published:** 2023-12-07

**Authors:** Zhao Xi, Ding Tong, Chang Honggang, Li Jinjin

**Affiliations:** 1 Research Institute of Natural Gas Technology, PetroChina Southwest Oil&Gasfield Company, Chengdu, Sichuan China; 2 National Energy R&D Center of High Sulfur Gas Exploitation, Chengdu, Sichuan China

**Keywords:** SO_2_ oxidation, Sulfur trioxide, Controlled condensation method, Tail gas treatment

## Abstract

The formation and emission of sulfur trioxide (SO_3_) in sulfur recovery unit has received increasing attention due to its adverse effects on the operation of plant and environment. Due to the excess oxygen, high concentration of SO_2_ and high temperature, SO_3_ formation in the sulfur recovery unit tail gas incinerator may significantly increase. A small horizontal tube reactor was employed to simulate the homogeneous oxidation of SO_2_ in the tail gas incinerator. The SO_3_ concentration was measured with a controlled condensation method at the outlet of the reactor. The present work focuses on the gas-phase chemistry and examines the impact of different combustion parameters and atmospheres on the formation of SO_3_ in the tail gas incinerator. Experiment results show that the increased O_2_ and SO_2_ concentrations along with increasing temperature are favorable for enhancing SO_3_ formation over the range of tested parameters. The presence of water vapor has an enhancing effect of SO_2_ oxidation in the experiments conducted. No significant effect of CO_2_ was found to the oxidation of SO_2_.

## Introduction

The oxidation of SO_2_ relate to the sulfur compounds combustion becomes an increasing concern. The high concentration of oxidation products such as sulfur trioxide (SO_3_), which could react with water molecule form H_2_SO_4_ or sulfuric acid mist, is not only a corrosion risk to equipment but also an environmental concern. In sulfur recovery unit, the tail gas stream from Claus tail gas treatment plant is predominantly N_2_, H_2_O vapor and CO_2_ depending on the original acid gas. This stream will also contain a number of residual sulfur compounds such as H_2_S, SO_2_, COS, CS_2_ and S vapor together with other components, such as CO and H_2_, at low concentrations. Compliance with regulatory guidelines of pollutant emission, requires the destruction of all reduced sulfur compounds in the tail gas down to low levels before allowing the stream to enter [1] the atmosphere [1, 2]. This is typically done by oxidizing the reduced sulfur compounds in the tail gas to SO_2_ within the plant incinerator. One of the preferred methods to incinerate the Claus tail gas stream is thermal incineration, where high temperature homogeneous gas phase reactions take place for oxidation of the reduced sulfur compounds and then all the sulphur compounds are converted to SO_2_. When excess oxygen is present and gas temperature is sufficiently high, a small amount of SO_2_ could be converted to SO_3_ in the flue gas [3–5]. SO_3_ is a great concern in anti-corrosion design, due to the presence of SO_3_ and vapor in the flue gas. Sulfuric acid can condense at very high temperature in flue gas, wherein the dew point of the sulfuric acid in the flue gas from the sulphur tail gas incinerator is typically 120–170 °C [6]. The Claus tail gas and sulphur degasser off-gases are both routed to the incinerator, resulting in relatively high concentration of SO_2_ in the flue gas. Due to the high concentration of O_2_, SO_2_ and water vapor in the incinerator, more SO_3_ tends to be formed. Higher SO_3_ concentration together with a mass of water vapor in the flue gas will obviously increase the acid dew point of the flue gas, increasing the risk of corrosion on the equipment. Care must be taken to prevent the steel shell of the equipment from getting below the dew point of the acid gas [7]. Beyond that, sulfuric acid in flue gas is an environmental concern, and a small amount of SO_3_ emission may cause serious environmental problems. With the decrease in temperature, the H_2_SO_4_ vapor condenses to form acid mist, leading to acid deposition [8], and furthermore, sulfuric acid mist is often the cause of the blue haze that often appears as the flue gas plume dissipates.

Many studies have been done to explore the formation of SO_3_ in combustion process by both experiment method and theoretical modeling [9–14]. In principle, SO_3_ formation from SO_2_ oxidation during the combustion process results from either homogeneous gas reactions or heterogeneous catalytic reactions. Sulfur trioxide is thermodynamically favored at lower temperatures, but kinetic limitations and short reaction time often prevent an SO_3_/SO_2_ partial equilibrium from being attained [10]. From the research work of Hindiyarti et al. [15] two reaction routes are recognized as the dominant pathways in the oxidation of SO_2_ in gas phase reaction.

The primary reaction is the direct reaction between SO_2_ and oxygen radical at temperature higher than 900 ℃:

1 $${\text{SO}}_{2} + {\text{O}}( + {\text{M}}) \Leftrightarrow {\text{SO}}_{3} ( + {M})$$

The other reaction path occurs under moist atmosphere, where oxidation of SO_2_ is strengthened in the presence of water vapor which increases the O/H radical concentration:

2 $${\text{SO}}_{2} + {\text{OH}}( + {M}) \Leftrightarrow {\text{HOSO}}_{2} ( + {M})$$

3 $${\text{HOSO}}_{2} + {\text{O}}_{2} \Leftrightarrow {\text{SO}}_{3} + {\text{HO}}_{2}$$

The first reaction path is the main source of SO_3_ formation at high temperature, and the second reaction path contributes mostly to the generation of SO_3_ under moist atmosphere at low temperature. HOSO_2_ is unstable at high temperature, and production of SO_3_ via HOSO_2_ is insignificant at temperatures of 1000 K and above [13, 16, 17].

Due to the significantly increasing concentration of SO_2_ and oxygen compared to air firing, lots of research works focus on the SO_3_ formation in oxy-fuel circulating fluidized bed [4, 14, 18–21]. SO_3_ concentration measurement under oxy-fuel condition also indicates an increase of SO_3_ concentration in flue gas [5]. Compared with the conditions in oxy-fuel combustion, because the Claus tail gas and sulphur degasser off-gases are both routed to the incinerator, the concentration of SO_2_ in tail gas incinerator are around 1% vol, higher than that in oxy-fuel combustion [14, 22]. Since limited work has been published focusing on the generation of SO_3_ in incinerators of the sulfur recovery unit, it is necessary to have a deep look at the formation of SO_3_ in the tail gas incinerator of the sulfur recovery unit.

In the present work, the focus is to investigate the homogeneous gas phase SO_3_ generation in the tail gas incinerator of the sulfur recovery unit under different combustion conditions. The effects of concentration of reactant gas, reaction temperature and the presence of impurities in flue gas were investigated on the formation of SO_3_.

## Experimental section

**Fig. 1:**
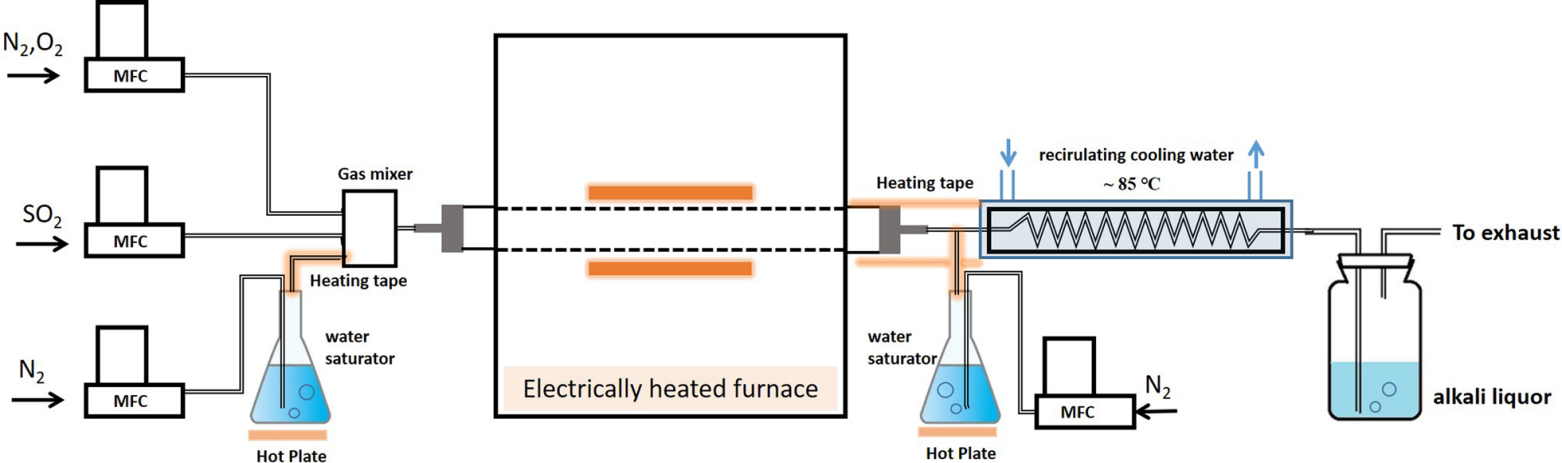
Experimental setup for homogeneous SO_2_ oxidation experiments

The experimental setup consists of a gas feeding system, a mixing chamber, a reactor system, an acid mist condenser and a tail gas treatment unit, as shown in Fig. 1. The gas feeding system consists of gas cylinders that are connected to the reactor system with mass flow controllers from the Beijing Sevenstar Electronics Co., Ltd. to the reactor system. High purity air, CO_2_, SO_2_, N_2_ (21% Oxygen in N_2_, 99.99% CO_2_, 10% SO_2_, 99.99%N_2_,) are used to simulate the reaction gas, N_2_ is the balance gas. The concentration of O_2_, SO_2_, CO_2_ in the reaction gas are adjusted by controlling the flowrate of corresponding mass flow controller. The controllers are adjusted before the experiment using a bubble flow meter. All the reaction gases including water vapor are mixed in the mixing chamber. A water saturator maintained at a controlled temperature was used to introduce water vapor into the N_2_ carrier gas. The amount of water vapor introduced to the reaction system is measured by the loss of weight of evaporated water. The concentration of water vapor is controlled by the flowrate of carrier gas. According to the actual condition in the tail gas incinerator of the sulfur recovery unit, the concentration of SO_2_ and oxygen in the tube reactor were set to be around 1% vol and 4% vol respectively. The reactor system consists of a quartz glass tube flow reactor that is inserted for the oxidation of SO_2_ [11]. The tube reactor characterized with 16 mm inner diameter and 60 cm total length, is located in a one zone electrically heated horizontal furnace with an isothermal length of 18 cm. According to the residence time of flue gas in tail gas incinerator, the residence time of reaction gas in the isothermal zone was set to be 2 s at the actual reaction temperature, and the total gas flow rate into the reactor was adjusted to meet the residence time, typically 263 ml/min (@1 atm, 273 K) for the reaction temperature at 1123 K. The temperatures in the tube reactor were measured under inert atmosphere with a type K thermoelectric couple (± 5 K). The gas line behind the outlet of reactor was electrically heated with a heating tape to a temperature around 250 ℃ for the purpose of avoiding condensation [10].

To determine the oxidation of SO_2_ in the reactor, the controlled condensation method (CCM) was employed to measure the concentration of SO_3_ at the outlet of tube reactor. SO_3_ was extremely reactive in flue gas, with the decrease of flue gas temperature, and water vapor and SO_3_ beginning to form vapor sulfuric acid. When the temperature of flue gas below is 200 ℃, almost all the SO_3_ is converted to H_2_SO_4_ in the humid condition of typical flue gas [23]. With the continuous decrease of flue gas temperature, once the temperature was below acid dew point, the gaseous H_2_SO_4_ began to condense to sulfuric acid. The temperature of gas line between the outlet of the tube reactor and the inlet of the condenser was controlled around 250 ℃ to avoid condensation. The temperature of flue gas in the condenser was set around 85 ℃ which was above the dew point of water vapor to prevent the condensation of water and associated SO_2_ capture by condense water, but also kept below the acid dew point which allow the condensation of sulfuric acid. With this approach, SO_3_ in the flue gas was selectively condensed and the interference of high concentration of SO_2_ in the flue gas is avoided. Moreover, about 0.5 g quartz wool is placed inside the condenser to improve the recovery rate of acid mist. When exploring the reaction between SO_2_ and O_2_, water vapor is needed to condense the SO_3_ in the flue gas, as shown in Fig. 1. A three-way valve was used at the outlet of the tube reactor for introducing water vapor into the flue gas, the same as the introducing of water vapor into the tube reactor. Furthermore, the total gas flow rate into the condenser can be adjusted through the three-way valve. All the experiments in this study, the total gas flow rate is set to be 1.5 L/min. Two measurements are conducted under one experiment condition, the average value of measurements is used as one data point.

The sulfuric acid present in the condenser was flushed by a known quantity of distilled deionized water. The conversion of SO_2_ to SO_3_ in the reactor was determined by the amount of H_2_SO_4_ in the condenser. An ion chromatogram analyzer (ICS-1100, Dionex) was used to determine the concentration of H_2_SO_4_ in the distilled deionized water. The measurement error of ion chromatogram analyzer is within 5%.

**Fig. 2:**
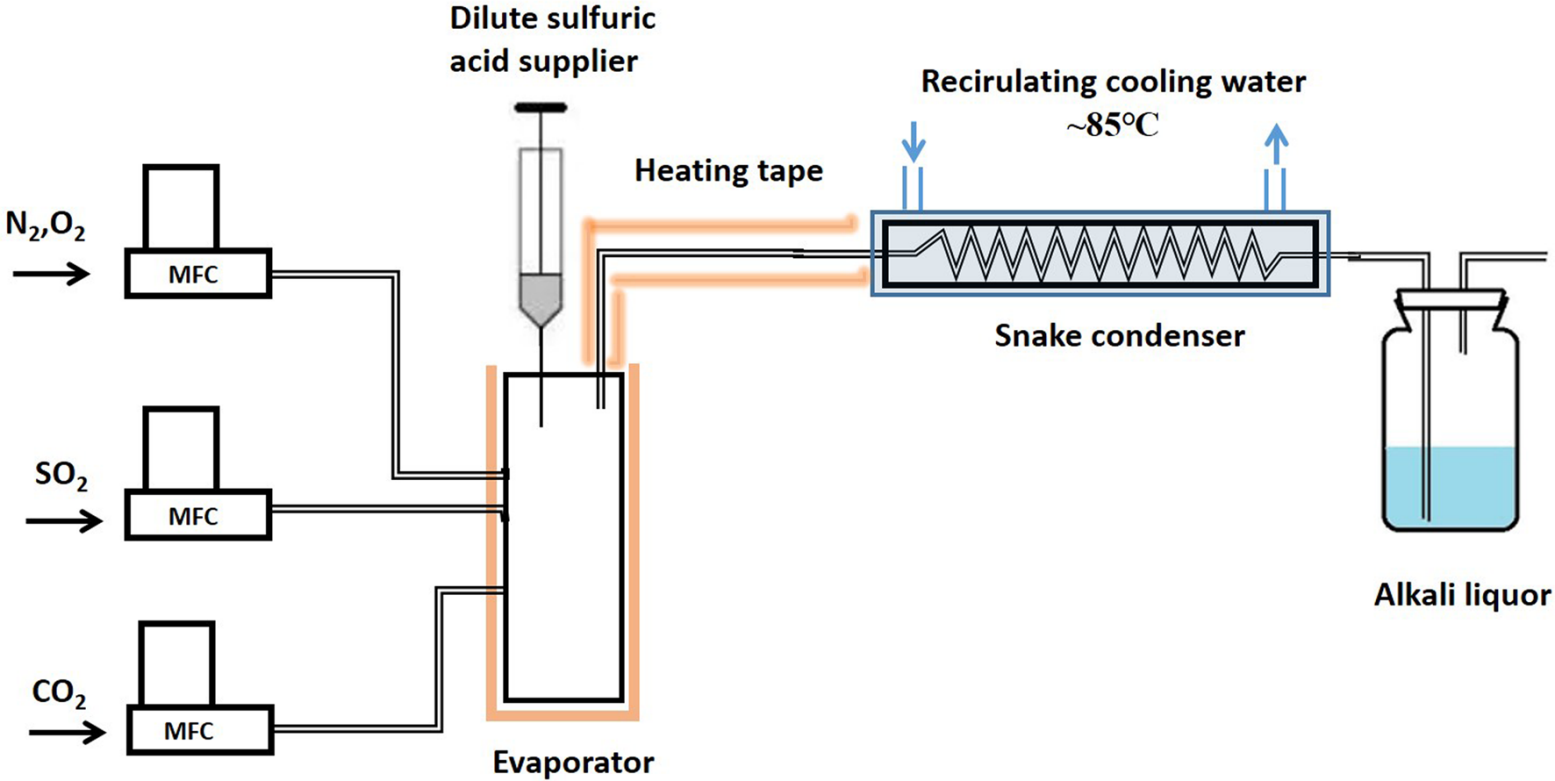
The diagram of SO_3_ collecting system built in laboratory

To evaluate the accuracy of the SO_3_ collecting system, a SO_3_ sampling system has been built in laboratory, as shown in Fig. 2, where dilute sulfuric acid has been evaporated to simulate the sulfuric acid mist in the reaction gas. In general, almost all the SO_3_ convert to sulfuric acid mist in the snake condenser. Therefore, the concentration of SO_3_ seem same as the concentration of sulfuric acid mist. The collecting efficiency of the condenser is defined as the ratio between the amount of sulfuric acid mist collect in the condenser and the amount of sulfuric acid introduced to the evaporator.

**Fig. 3:**
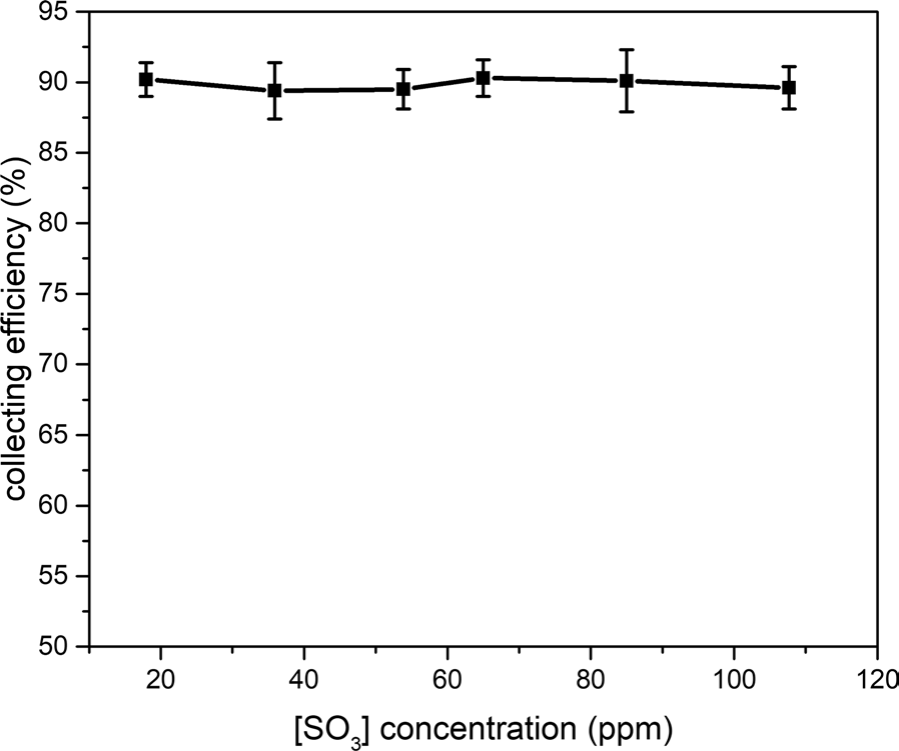
The collecting efficiency of SO_3_ collecting system with different SO_3_ concentration

In the sampling experiments, the concentration of SO_2_, O_2_ and CO_2_ in the gas is set to be 1%, 4% and 20%, N_2_ is the balance gas. The flowrate of the gas is set at 1.5 L/min, temperature of evaporator is set around 400 ℃, the temperature of heating tape and condenser is set to be 250 and 85 ℃, the flow rate of dilute acid introduced into the evaporator is set at 0.24 ml/min. The adjustment of the SO_3_ concentration in the gas could be realize by changing the concentration of dilute acid introduced to the system. The concentration of dilute acid introduced into the evaporator is about 5 mM to 30 mM. The average value of two measurements under one experiment condition is used as one data point. As shown in Fig. 3, the collecting efficiency of sampling system is relatively stable with the change of SO_3_ concentration, the collecting efficiency is around 90%.

## Results and discussion

### Effect of SO_2_ concentration

To study the effect of input SO_2_ concentration on the output SO_3_ concentration in the flue gas, the conversion of SO_2_ to SO_3_ and the order of reaction with respect to the SO_2_ concentration were obtained. Experiments were conducted at temperature 850 ℃ with a fixed oxygen concentration of 4 vol% and by varying the SO_2_ concentration among 0.52, 0.75, 0.87, and 1.31 vol%.

When the compositions in the reaction are far away from equilibrium, the reversible reaction can be ignored, and under such conditions, the rate of the generation of SO_3_ can be expressed by the following empirical equation:

4 $$r_{{{\text{so}}_{3} }} = k_{f} {\text{C}}_{{{\text{SO}}_{2} }}^{n} {\text{C}}_{{{\text{O}}_{{2}} }}^{m} = k_{f} P_{{{\text{SO}}_{{2}} }}^{n} P_{{{\text{O}}_{{2}} }}^{m} /[RT]^{m + n}$$

where n and m are reaction orders with respect to SO_2_ and O_2_ concentrations. The oxidation of SO_2_ was less than 2% under our experiment conditions, its concentration can be assumed to be constant (i.e.,$P_{{{\text{SO}}_{{2}} }}$ = $P_{{{\text{SO}}_{2} }}^{0}$). Additionally, the consumed oxygen calculated from the generated SO_3_ were calculated to be less than 1%. Therefore, for the sake of simplicity, if it is assumed that both the reactants were in excess compared to the product, the relationship ${\text{ln}}(P_{{{\text{so}}_{3} }} ) = \ln (k{\prime} t_{res} ) + m\ln (P_{{{\text{so}}_{2} }}^{0} )$ can be derived, where k´ is equal to k_f_ times $(P_{{{\text{O}}_{2} }}^{0} )^{{\text{n}}} /[RT]^{{{\text{m}} + n - 1}}$. Hence, the slope (m) of a plot of ${\text{ln}}(P_{{SO_{3} }} )$ versus ${\text{ln}}(P_{{{\text{SO}}_{2} }}^{0} )$ would result in the order of reaction with respect to the SO_2_ concentration.

**Fig. 4:**
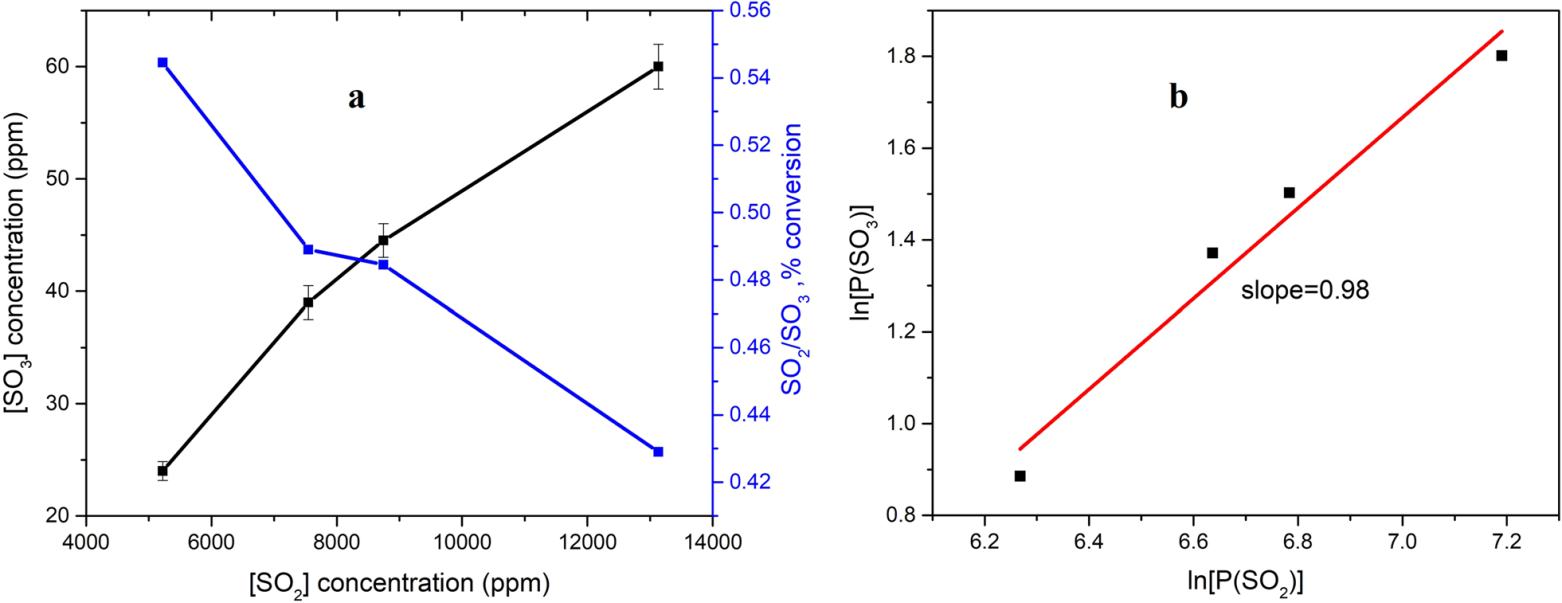
**a** Effect of the input SO_2_ concentration on the output SO_3_ concentration in the flue gas and conversion of SO_2_ to SO_3_, **b** order of dependency with respect to SO_2_ concentration for SO_3_ formation reaction. Inlet gas mixture for the experiments: variable ppm of SO_2_, 4 vol% O_2_, and balance N_2_, at temperature of 850 ℃

In Fig. 4a, the concentration of SO_3_ at the outlet of the reactor and the conversion of SO_2_ is plotted against the input SO_2_ concentration. SO_2_ concentration varied from 5200 ppm to 13,100 ppm, corresponding to the high SO_2_ concentration in the tail gas incinerator. As expected, the concentration of SO_3_ was higher with the increasing SO_2_ concentration in the flue gas. The concentration of SO_3_ at the outlet of the reactor was measured from 24 to 60 ppm in the experiments conducted. The conversion of SO_2_ to SO_3_ ranged from 0.544% for 5200 ppm of SO_2_ input to 0.489% for 7548 ppm of SO_2_, 0.484% for 8747 ppm of SO_2_, and 0.429% for 13,100 ppm of SO_2_.

The oxygenation efficiency varied with the concentration of SO_2_ for the SO_2_ oxidation are according with previous works [10, 24]. It's worth noting that from Fig. 4a, with the increase of SO_2_ concentration in the flue gas, a decrease of SO_2_ conversion is observed, which is consistent with the conclusion in the previous studies [5, 10, 12, 24]. As shown in Fig. 4b, ${\text{ln}}(P_{{{\text{SO}}_{3} }} )$ is plotted against $\ln (P_{{{\text{SO}}_{2} }}^{0} )$, the linear fitting of the data point results in a slope of 0.98 under the conducted experimental parameters. The slope is close to unity which implies that the order of the reaction with respect to the SO_2_ concentration is unity. In the previous work of Forzatti et al., where the oxidation of SO_2_ was explored on a honeycomb catalyst, the results indicated that a first-order dependence could give a reasonable approximation of the SO_2_ oxidation in the range from 0 to 1000 ppm of SO_2_ [25, 26]. The reaction order obtained in present study is the SO_2_ oxidation taking place in gas phase, which is different from the previous studies [25, 26].

### Effect of O_2_ concentration

The effect of O_2_ concentration on the SO_2_ oxidation in gas phase was studied in a manner similar to that employed for studying the effect of the SO_2_ concentration on the SO_3_ formation. The conversion of SO_2_ to SO_3_ and the order of reaction with respect to the O_2_ concentration were obtained. According to combustion condition in the tail gas incinerator of the sulfur recovery unit, experiments were performed at temperature 850 ℃ with a fixed SO_2_ concentration of 1 vol% and by varying the O_2_ concentration in 1, 2, 3, 4 and 5 vol%.

**Fig. 5:**
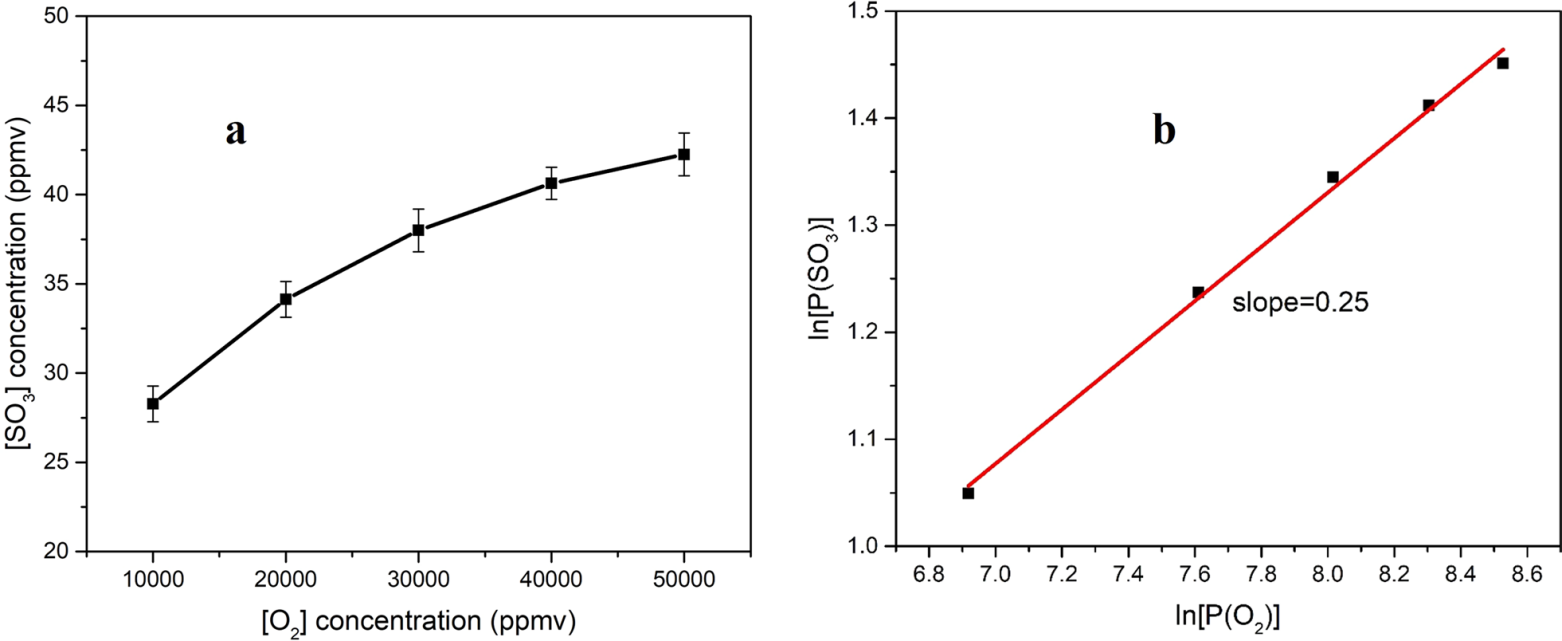
**a** Effect of the input O_2_ concentration on the output SO_3_ concentration in the flue gas, **b** order of dependency with respect to O_2_ concentration for SO_3_ formation reaction. Inlet gas mixture for the experiments: variable ppm of O_2_, 1 vol% SO_2_, and balance N_2_, at temperature of 850 ℃

As shown in Fig. 5a, the concentration of SO_3_ at the outlet of the reactor is plotted against the input O_2_ concentration. As anticipated, the oxidation of SO_2_ is enhanced with the enrichment of oxygen, due to more molecular oxygen existing in the flue gas. As O_2_ concentration increases from 1 vol% to 2 vol%, the concentration of SO_3_ increases significantly from 28 to 34 ppm. Further increasing O_2_ concentration from 4 vol% to 5 vol% does not result in significant increase of SO_3_ formation, which is consistent with the previous work from Fleig et al. [4]. Different form the statement from previous work that at least 1% excess O_2_ is needed for SO_2_ oxidation [27], the conversion of SO_2_ in the atmosphere of low excess O_2_ is observed. In Fig. 5b, ${\text{ln}}(P_{{{\text{SO}}_{3} }} )$ is plotted against $\ln (P_{{{\text{O}}_{{2}} }}^{{0}} )$, and the straight line fitted through the data points results in a slope of 0.25 under the conducted experimental conditions. According to the slope of the fitting data, the reaction order for the SO_2_ oxidation with respect to the O_2_ is 0.25, which is similar to the research of Fan et al. [28]. The fractional order implies that the reaction between SO_2_ and O_2_ is not an elementary reaction.

### Effect of experimental temperature

**Fig. 6:**
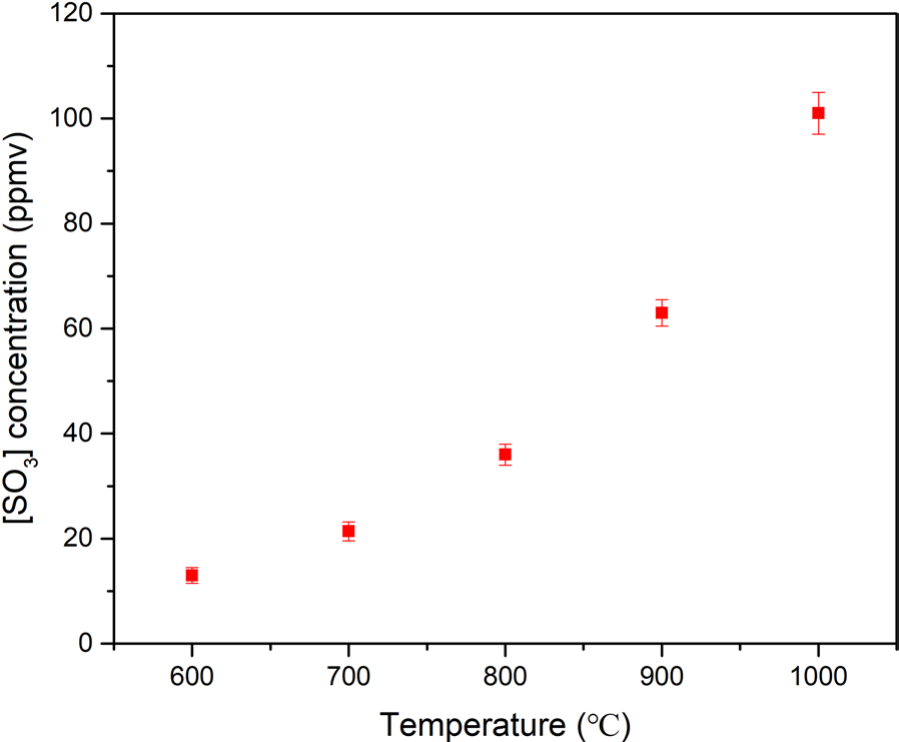
Effect of the experiment temperature on the output SO_3_ concentration in the flue gas. Inlet gas mixture for the experiments: 4 vol % of O_2_, 1 vol % SO_2,_ and balance N_2_

Under the standard-state conditions (i.e., 25 ℃ and 1 atm), the change in Gibbs free energy of the reaction 1 is − 71 kJ/mol [29]. Therefore, thermodynamically, the oxidation of SO_2_ in gas phase is feasible. However, the chemical kinetics of reaction 1 limits the conversion of SO_2_. The oxidation of SO_2_ is too slow at low temperature. According to the thermodynamic model [18], thermodynamically, complete oxidation of SO_2_ is expected at below 500 °C and, as the temperature is increased to 900 and 1000 °C, the conversion decreased to about 6.75 and 3.21%, respectively. To get a deeper insight into the temperature effect, the temperature dependence of the oxidation of SO_2_ to SO_3_ was investigated using a reactant mixture consisting of 1 vol % SO_2_, 4 vol % O_2_ and by varying the experiment temperature among 600, 700, 800, 900 and 1000 ℃. Figure 6 shows the result of the temperature variation, and a positive correlation was found between the SO_2_ oxidation and temperature. The concentration of SO_3_ in the flue gas increased from 13 to 101 ppm as the temperature increased from 600 to 1000 ℃ in the performed experiments. It's worth noting that water vapor is absent in the tube reactor in the experiments conducted, and the O radicals combine with SO_2_ corresponding to the oxidation of SO_2._ The conversion of SO_2_ is 0.13% at 600 ℃.

Higher concentration of O radicals at high temperature contributes to the SO_2_ oxidation which results in conversion of 1% at 1000 ℃. This is consistent with the finding by Bayless [27].

### Effect of water vapor

**Fig. 7:**
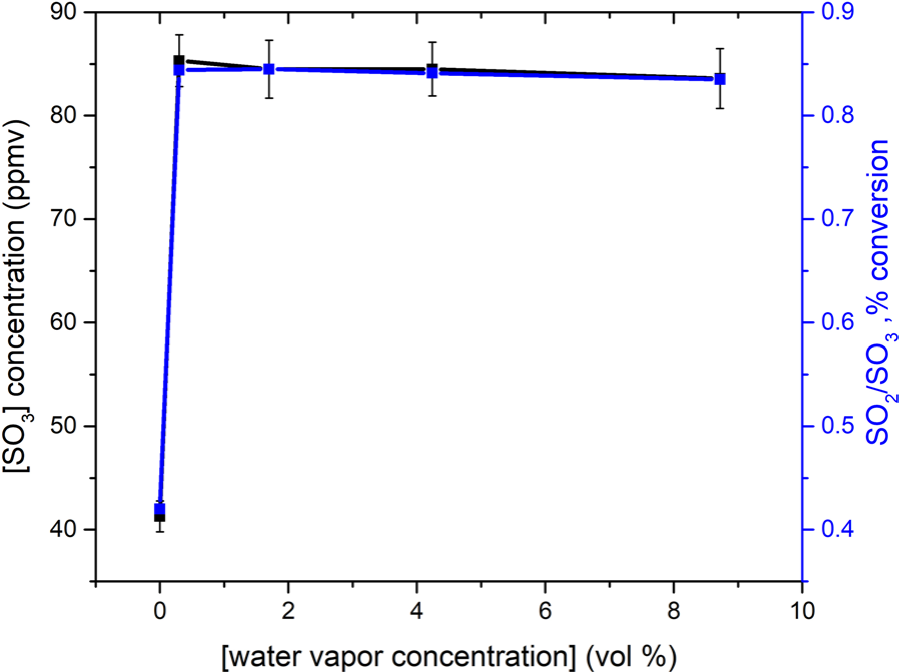
Effect of the input moisture on the output SO_3_ concentration in the flue gas and the conversion of SO_2_. Inlet gas mixture for the experiments: variable concentration of H_2_O, 1 vol% SO_2_, 4 vol% O_2_ and balance N_2_, at temperature of 850 ℃

Generally speaking, the presence of moisture is inevitable in the flue gas in the tail gas incinerator. To evaluate the effect of moisture content on the oxidation of SO_2_ in gas phase, the concentration of SO_3_ at the outlet of the reactor was measured at water vapor contents varying form 0 vol% to 8.7 vol% in the flue gas. As shown in Fig. 7, the introducing of moisture in the reactor results in a significantly enhance of SO_2_ oxidation. The SO_3_ concentration at the outlet of the reactor is increased from 41 to 85 ppmv with the presence of water vapor.

5 $${\text{HO}}_{2} ( + M) \Leftrightarrow {\text{H}} + {\text{O}}_{2}^{{}} ( + M)$$

6 $${\text{H}} + {\text{O}}_{2} \Leftrightarrow {\text{O}} + {\text{OH}}$$

7 $${\text{H}}_{2} {\text{O}} + {\text{O}} \Leftrightarrow {\text{OH}} + {\text{OH}}$$

The water vapor exist in the flue gas increases the formation of H radicals through reaction 5 and thereby the formation of O and OH radicals by reaction 6. Furthermore, the concentration of OH radicals is increased through reaction 7. The increasing concentration of OH radicals enhances the oxidation of SO_2_ by reaction 2. However, in the range of moisture content from 0.3% to 8.7%, all the measurements show comparable values, and no obvious dependency of the SO_2_ oxidation on the water vapor content is visible. In the literature from Tobias and Belo [10, 24], a similar trend was observed with the increase of moisture content. It's worth noting that due to the need to condense the SO_3_ in the flue gas, all the experiments in the reference literature were conducted with the presence of moisture in the reactor. Due to the introducing of water vapor at outlet of the reactor in the present work, the experiments performed under conditions without water vapor in the reactor are feasible. The obvious enhancing effect of water vapor for SO_2_ oxidation in gas phase was demonstrated in this study. Further work is needed to elucidate the independence of the varying moisture content for the SO_2_ conversion in gas phase.

### Effect of CO_2_

**Fig. 8:**
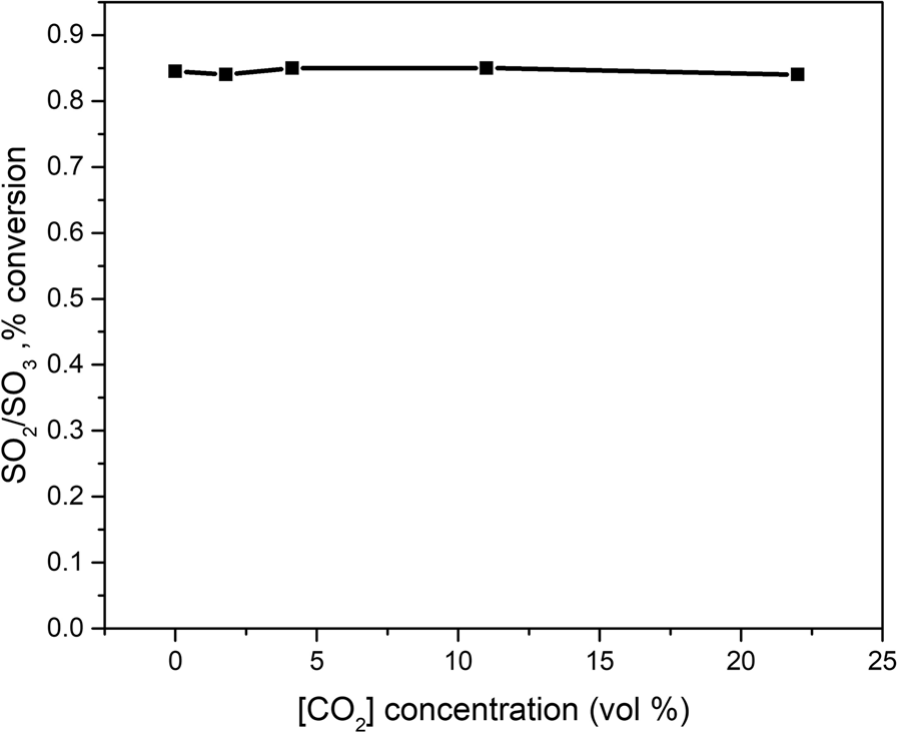
Effect of the input CO_2_ on the conversion of SO_2_. Inlet gas mixture for the experiments: variable concentration of CO_2_, 1 vol% SO_2_, 4 vol% O_2_, 3.8% moisture and balance N_2_, at temperature of 850 ℃

Due to the considerable partial pressure of CO_2_ in the tail gas incinerator of the sulfur recovery unit, the effect of CO_2_ on the oxidation of SO_2_ is considered. Figure 8 shows the SO_2_ conversion varying with CO_2_ concentration. As can be seen in Fig. 8, the increasing content of CO_2_ has a very slight effect on the SO_2_ oxidation at experimental temperature. The presence of CO_2_ can depress the availability of O/H radicals, since the reaction between CO_2_ and H radicals, as shown in reaction 8, is in competition with reaction 6. Therefore, the concentration of H radical can be decreased in the presence of high CO_2_ concentration. The H radical is important to the production of O radicals and OH production. Thus, the SO_2_ conversion would be decreased in the presence of CO_2_. However, on the other hand, the third body efficiency of reactions 1 and 2 for CO_2_ is higher than that for N_2_, which counteracts the depressed effect occurring through radical removal by CO_2_ [30]. To conclude, the presence of CO_2_ in the flue gas influences the SO_2_ oxidation in both directions (production/reduction). Overall, no significant effect of CO_2_ was found to the oxidation of SO_2_.This result is consistent with previous [12] works [5].

8 $${\text{CO}}_{2} + {\text{H}} \Leftrightarrow {\text{CO}} + {\text{OH}}$$

## Conclusions

The homogeneous oxidation of SO_2_ to SO_3_ was investigated using a quartz flow reactor under the combustion conditions similar to those in the tail gas incinerator of the sulfur recovery unit. The conversion of SO_2_ decreased with the increase of SO_2_ concentration. The reaction order of SO_3_ formation was determined to be 0.98 and 0.25 with respect to SO_2_ and O_2_. The SO_2_/SO_3_ conversion increased from 0.13% to 1% as the temperature increased from 600 to 1000 ℃. The enhancing of SO_2_ conversion is the result of higher concentration of O radicals at high temperature. The presence of water vapor has a significant effect to promote the conversion of SO_2_. SO_2_ oxidation was also found to be independent of the CO_2_ in the flue gas.

Experiments were designed to determine the effect of different combustion conditions on SO_3_ formation. The results of this research would provide an idea to reduce the production of SO_3_ in tail gas combustor (such as the Claus installations). In the future, the effect of the combustion chamber structure combine with the operating parameter of combustor on the SO_3_ formation maybe the research direction.

## Data Availability

All data generated or analysed during this study are included in this published article.
